# Meals, Microbiota and Mental Health in Children and Adolescents (MMM-Study): A protocol for an observational longitudinal case-control study

**DOI:** 10.1371/journal.pone.0273855

**Published:** 2022-09-01

**Authors:** Birna Asbjornsdottir, Bertrand Lauth, Alessio Fasano, Inga Thorsdottir, Ingibjorg Karlsdottir, Larus S. Gudmundsson, Magnus Gottfredsson, Orri Smarason, Sigurveig Sigurdardottir, Thorhallur I. Halldorsson, Viggo Thor Marteinsson, Valborg Gudmundsdottir, Bryndis Eva Birgisdottir

**Affiliations:** 1 Faculty of Medicine and Health Science Institute, University of Iceland, Reykjavik, Iceland; 2 Faculty of Food Sciences and Nutrition and Health Science Institute, University of Iceland, Reykjavik, Iceland; 3 Mucosal Immunology and Biology Research Center, Massachusetts Hospital *for* Children, Harvard Medical School, Boston, Massachusetts, United States of America; 4 Department of Child and Adolescent Psychiatry (BUGL), Landspitali University Hospital, Reykjavik, Iceland; 5 Faculty of Pharmaceutical Sciences and Health Science Institute, University of Iceland, Reykjavik, Iceland; 6 Department of Science, Landspitali University Hospital, Reykjavik, Iceland; 7 Department of Infectious Diseases, Landspitali University Hospital, Reykjavik, Iceland; 8 Faculty of Psychology and Health Science Institute, University of Iceland, Reykjavik, Iceland; 9 Department of Immunology, Landspitali University Hospital, Reykjavik, Iceland; 10 Matís ohf., Microbiology Group, Reykjavík, Iceland; 11 Icelandic Heart Association, Kopavogur, Iceland; Kwantlen Polytechnic University Faculty of Science and Horticulture, CANADA

## Abstract

Recent studies indicate that the interplay between diet, intestinal microbiota composition, and intestinal permeability can impact mental health. More than 10% of children and adolescents in Iceland suffer from mental disorders, and rates of psychotropics use are very high. The aim of this novel observational longitudinal case-control study, “Meals, Microbiota and Mental Health in Children and Adolescents (MMM-Study)” is to contribute to the promotion of treatment options for children and adolescents diagnosed with mental disorders through identification of patterns that may affect the symptoms. *All* children and adolescents, 5–15 years referred to the outpatient clinic of the Child and Adolescent Psychiatry Department at The National University Hospital in Reykjavik, Iceland, for one year (*n≈1*50) will be invited to participate. There are two control groups, i.e., sex-matched children from the same postal area (*n≈1*50) and same parent siblings (full siblings) in the same household close in age +/- 3 years (*n*<150). A three-day food diary, rating scales for mental health, and multiple questionnaires will be completed. Biosamples (fecal-, urine-, saliva-, blood samples, and buccal swab) will be collected and used for 16S rRNA gene amplicon sequencing of the oral and gut microbiome, measurements of serum factors, quantification of urine metabolites and host genotype, respectively. For longitudinal follow-up, data collection will be repeated after three years in the same groups. Integrative analysis of diet, gut microbiota, intestinal permeability, serum metabolites, and mental health will be conducted applying bioinformatics and systems biology approaches. Extensive population-based data of this quality has not been collected before, with collection repeated in three years’ time, contributing to the high scientific value. The MMM-study follows the “Strengthening the Reporting of Observational Studies in Epidemiology” (STROBE) guidelines. Approval has been obtained from the Icelandic National Bioethics Committee, and the study is registered with Clinicaltrials.gov. The study will contribute to an improved understanding of the links between diet, gut microbiota and mental health in children through good quality study design by collecting information on multiple components, and a longitudinal approach. Furthermore, the study creates knowledge on possibilities for targeted and more personalized dietary and lifestyle interventions in subgroups.

**Trial registration numbers**: VSN-19-225 & NCT04330703.

## Introduction

### Mental disorders and intestinal permeability

It has been estimated that up to 20% of children and adolescents worldwide experience mental disorders [1]. Symptoms from the gastrointestinal (GI) tract are common among children with mental disorders [2–4]. Inherent to the rationale of a gastrointestinal role in mental disorders, including imbalanced microbiota, is the idea of intestinal permeability (IP) of the intestinal epithelial barrier. Imbalanced microbiota can result in dysbiosis, which, if left untreated, can affect the intestinal epithelial paracellular pathway [5]. Increased IP allows food-borne chemicals, including toxins, and microbial components, to enter tissues beneath the intestinal lining, which can result in local and systemic inflammation caused by pro-inflammatory cytokines in the intestine as well as outside the intestinal tract [6]. Loss of intestinal barrier function may lead to neuroinflammation and neuroimmune disorders such as autism spectrum disorder (ASD) [1, 4, 7], chronic fatigue syndrome [8], major depressive disorders (MDDs) [9, 10], and schizophrenia [11, 12].

Zonulin, a family of structurally and functionally related proteins, is currently the only known physiologic modulator of intercellular tight junctions and affects mechanisms that regulate the intestinal epithelial paracellular pathway. Zonulin upregulation has been associated with several chronic inflammatory diseases, low-grade inflammation, and autoimmune diseases, including ASD and attention deficit hyperactivity disorder (ADHD), which might have an autoimmune component [1, 13]. There is growing evidence, in genetically susceptible individuals, on changes in the intestinal microbiome composition and function, i.e., intestinal dysbiosis, possibly causing functional changes in intestinal permeability (IP). Functional changes in IP may lead to increased IP, contributing to increased antigen trafficking and break of tolerance, increasing the probability of developing a chronic inflammatory disease [6, 14, 15].

Intestinal microbiota-derived antigen and endotoxin trafficking from the lumen to the lamina propria has been found to trigger innate-and immunoregulatory responses, which may cause a pro-inflammatory micro milieu. Over time, an adaptive immune response could be mounted, producing pro-inflammatory cytokines, which may cause further opening of the paracellular pathway for the passage of antigens. The specific host genetic background has been found to affect where the inflammatory process will be targeted, i.e., in which organ or tissue [6, 13, 14, 16–18]. Dysbiosis and the presence of inflammation, as well as neuroinflammation, have all been described in mental disorders [13, 19].

The gut-brain axis involves bidirectional communication between the central and the enteric nervous system [20]. Increased IP might affect the enteric nervous system, impacting both mood and behavior [12]. The emerging recognition of the relationship between the gut-brain axis and the neuro-immune system provides a novel approach for potentially better understanding and managing mental disorders. There is an urgent need to investigate these emerging interrelations further.

### Mental disorders in children

The etiology of most mental disorders is unknown, but both genetic and environmental factors seem to play a role [21, 22]. Many mental disorders become visible during childhood and pose a challenge both to the children and their families, affecting everyday life and wellbeing. Research shows that up to 20% of children and adolescents may experience behavioral, mental, and neurodevelopmental disorders, including 10% with clinical diagnosis, resulting in disability in young people worldwide [23–25]. The most common mental disorders are ADHD, anxiety disorders, ASD, eating disorders, depressive disorders, disruptive disorders, tic disorders (TD) [26], and comorbidities are common. In the Diagnostic and Statistical Manual of Mental Disorders, 5th edition (DSM-V), neurodevelopmental disorders (ND) include ADHD, ASD, and TD. These disorders should be viewed as a spectrum rather than distinct disorders due to shared symptoms, genetics, and neuropathology [23, 27].

Recent studies that follow children from birth to adulthood indicate that most adult mental health disorders begin its origin in early childhood and adolescence [28, 29]. Despite evidence suggesting that overdiagnosis and overtreatment might exist to some extent, rates of diagnoses have increased substantially. A growing number of children and adolescents are now requiring treatments, including many types of interventions. In a systematic review of the literature that included 41 studies conducted in 27 countries from every world region [30], worldwide pooled prevalence of mental disorders was 13.4% (CI 95% 11.3–15.9). The most common diagnoses were: any anxiety disorder 6.5% (CI 95% 4.7–9.1), any depressive disorder 2.6% (CI 95% 1.7–3.9), ADHD 3.4% (CI 95% 2.6–4.5), and any disruptive disorder 5.7% (CI 95% 4.0–8.1). In comparison to the prevalence of other childhood chronic health conditions, such as obesity (16.8%) [31] and asthma (8.5%) [32], the high frequency of mental disorders and their associated negative consequences, render them major health priorities [30]. The burden of mental disorders among children aged 5–14 years has been studied in each of the six regions of the World Health Organization. Disability-adjusted life-years (DALYs) are the main indicator and are built from years of life lost (YLLs) and years of life lived with disability (YLDs). These studies showed that mental disorders in children are among the leading causes of YLDs and of DALYs in Europe and America [33].

A similar pattern is seen in Iceland at the outpatient Child and Adolescent Psychiatric Department (BUGL), where more than half of the children seen for the first time each year (*n≈*150) are diagnosed with ADHD, over 40% with anxiety disorders, over 30% with depressive disorders or disruptive disorders, and up to 25% with behavioral disorders or ASD, where overlapping of disorders is common [34, 35]. However, as mental disorders are thought to be products of multiple interacting causal factors rather than a single cause and are therefore multidimensional, mental disorders should preferably be evaluated dimensionally instead of categorically [36]. This has been introduced into the most recent Diagnostic and Statistical Manual of Mental Disorders (DSM-5), with more latitude to selecting severity of symptoms instead of a strictly categorical model where a disorder is either present or absent. In a research context, this approach is also considered more useful [37], especially as studies indicate that similar symptoms can have very different genetic origins [38, 39]. Clinical guidelines usually do not recommend medication as the first line of treatment for mental disorders except in severe cases [40]. Indeed, medication can result in many unwanted side effects. Other standard therapeutic options are psychological treatments [41, 42] depending upon the service available at each location. However, it is urgent to consider also other aspects. For example, symptoms from the GI tract are common among children with mental disorders [2–4], and there are indications that these have a higher prevalence of atopic reactions [43–45]. Research shows that childhood ADHD is associated with atopic diseases and impetigo [44]. Atopic diseases seem to be associated with ADHD symptoms severity in children and adults [46]. However, there are possible limitations inherent to observational studies and further research is needed. It is necessary to study the underlying mechanisms as well as to evaluate preventative, diagnostic and therapeutic interventions.

### Diet, intestinal microbiota, and mental disorders

In recent years, the association between diet, nutritional status, and the intestinal microbiota [47–49] has become a focus of interest, not least concerning mental health disorders [50–56]. The microbiota in children is affected by dietary habits and influences nutritional status. There is a link between the gut microbiota and malnutrition in children, as alteration in microbiota of severe acute malnourished children exhibits lower relative diversity when compared to healthy children. Moreover, the microbiota does include decreased levels of *Bacteroides* and increased levels of *Proteobacteria* in malnourished children [57]. The gut microbiota has been associated with obesity, a multifactorial chronic disorder, as is associated with other metabolic disorders and the gut microbiota is considered one of environmental regulators [58]. Several human and animal studies have revealed increased number of *Firmicutes* versus decreased number of *Bacteroidetes* in obesity [59–61]. Growth stimulation of the generally considered beneficial bacteria of Bifidobacterium and Lactobacillus by nutritional strategies with fiber or prebiotics may impact gastrointestinal health [62]. The microbiota can thus be affected by both pre- and probiotics [63–66] while dietary factors have been associated with both mental health and preexisting, existing, or other GI health problems [4, 67–69]. Furthermore, studies have indicated the importance of a healthy diet, i.e., healthy diet reflecting consumption of salads, fruit, fresh vegetables, and fish and the effects of whole-of-diet interventions rather than examining only individual foods/nutrients, as a potential factor in the treatment of anxiety [20], depression [70–72], and ADHD [73, 74]. In addition, single nutrients, e.g., optimal intake of vitamin D, omega-3 fatty acids [75–78] and iron and low exposure to heavy metals show positive outcome [79]. However, vitamin D, iron and low exposure to heavy metals did neither improve anxiety or depression except for omega-3 fatty acids which improved anxiety [78].

Studies have provided substantiated evidence in support of docosahexanoic acid (DHA), an omega-3 fatty acid, being a beneficial bioactive compound for brain function but it’s possible effects on depression disorders are still unclear [80]. Preliminary data point towards beneficial effects of omega-3 fatty acids on pediatric depression [81–86]. However, sufficient evidence is lacking to support/rule out that omega-3 fatty acids are potential treatments for depression as large-scale studies are needed to validate these results.

It is well known that eating is an integral part of the behavioral challenges in many mental disorders, with monotonic food choices and food aversions being common, which in turns affects the microbiota [87, 88]. The intestinal microbiota among children with disorders such as ASD and ADHD has been found to have a different composition compared to both community controls and healthy full siblings [89–93] living in the same household. The studies reveal a significant increase in the Bacteroidetes/Firmicutes ratio and *Clostridium* levels in children diagnosed with ASD and reduced *Bifidobacterium* levels in children diagnosed with ASD or ADHD. However, these studies often include few participants as well as being methodologically heterogeneous, making it hard to generalize the results [94–96].

The symbiotic relationship with the microbiota has evolved, and studies indicate that the host’s genetic composition might be related to host-microbiota composition [97]. Research has shown that the intestinal microbiota might influence the host genomic organization and gene regulation, i.e., gene expression in the gastrointestinal tract, and the mechanisms of gene regulation in cells of the immune system [98]. Cell components from the microbiota including their metabolites seem to be involved in the transcriptional response of the host to microbial colonization [99, 100]. Research shows that the intestinal microbiome may remodel host chromatin, cause differential splicing, directly interrupt host signaling cascades, and alter epigenetic landscape [101]. However, mechanistic understanding of this relationship is still in its infancy, although the phenomenology of the cross-regulation of gene expression between the intestinal microbiota and the cells of the immune system has been established [102]. Host genetics may differentially influence distinct members of the microbiota. Research has shown that abundances of certain taxa is different between monozygotic twin pairs when compared with dizygotic twin pairs [98].

Furthermore, genome-wide association studies have identified numerous single-nucleotide polymorphisms (SNPs) associated with mental disorders [103], which can be used to calculate individual polygenic risk scores for study participants. However, these SNPs are associations and mechanisms are still unknown.

Microbial imbalance, i.e., intestinal dysbiosis can be defined as qualitative and quantitative changes in the intestinal microbiota, the local distribution, and metabolic activity [5]. Dysbiosis has long been implicated in the development or exacerbation of mental disorders [104]. It has been suggested that the intestinal microbiota and the brain are linked in a bidirectional relation, i.e., the microbiome–gut–brain axis [105]. The advances in research and in understanding of the role of the microbiome in neurodevelopment and mental disorder have been remarkable in the last few years. Influences of the microbiome and how it might contribute to dysregulation of brain function is an area of growing interest [52, 106–110]. Diet-induced dysbiosis substantially influences brain function through shaping the intestinal microbiome [104, 105, 111].

Studies have also established a potential link between salivary microbiome alterations and disease initiation [112–114]. Wingfield et al. [113] revealed that the composition of the oral microbiome is associated with depression in young adults and Quing et al. [114] provided hypothesis of how the oral microbiota could influence schizophrenia. Differences in alpha and beta diversity of the salivary microbiome have been observed in both these studies, with clear separation into distinct clusters between cases and healthy controls [113, 114]. More research is needed on the oral-brain axis to clarify the biological links and interconnections between the oral microbiome and the pathophysiology of mental disorders.

Unhealthy dietary pattern may increase the risk of developing depression or anxiety, whereas a healthy dietary pattern may decrease it [105]. Diet is one of the most significant factors that influences the human intestinal microbiota structure and function, and the association found between diet and mental health might partially be through the microbiota. An adequate supply of micronutrients and macronutrients is essential to well-being and provides the foundation for microbiome health, low inflammation as well as for the efficacy of other psychotherapeutic and psychopharmacological interventions [115, 116].

Dietary effects on the gut microbiome are evident, even as early as infancy. Intestinal microbiome differences have been observed between breast-fed infants with a greater prevalence of Bifidobacteria compared to formula-fed infants [117]. Existing human studies of depression and intestinal microbiota report depression-specific findings regarding proportions of microbiota [56, 118–120]. Moreover, research shows an enrichment of pro-inflammatory bacteria as well as a depletion of specific anti-inflammatory butyrate-producing bacteria in subjects diagnoses with anxiety, bipolar disorder, depression, and schizophrenia [121].

There is growing evidence on changes in the intestinal microbiota composition and function, i.e., intestinal dysbiosis, which may cause functional changes in intestinal permeability (IP) in genetically susceptible individuals. Functional changes in IP may lead to increased IP, which could contribute to increased antigen trafficking and break of tolerance, and therefore, increasing the probability of developing a chronic inflammatory disease [6, 14, 15].

Loss of intestinal barrier function may lead to subsequent increased serum levels of microbiota-derived molecules and, in turn, activation of the immune system, which may lead to neuroinflammation. This has been described in several neuroimmune disorders such as ASD [1, 4, 7], chronic fatigue syndrome [8], major depressive disorders (MDDs) [9, 10], and schizophrenia [11, 12].

It is vital to study the intestinal microbiota and IP further regarding diet and mental health [56]. It is important to overcome previous limitations and increase the number of subjects, coordinate strategies, and assess confounding factors, e.g., the severity of the mental disorders and problems from the GI tract, and secure quality collection [94, 122] as well as lifelong medication use. Biosamples will be collected and analyzed with the latest technology factors in addition to the microbiota, that can shed a better light on research questions and potential mechanisms. As data on the gut-brain axis demonstrates association between intestinal microbiota composition and mental disorders such as depression, we will collect fecal samples within this study for microbiome analysis. However, longitudinal studies are lacking, and therefore in this present study, collection of data will be repeated in three years. These are the basis of the current study on Meals, Microbiota and Mental Health in Children and Adolescents, i.e., the MMM study.

Our hypothesis for the current study is the following; There are differences between cases and controls regarding dietary intake, nutritional status and/or the intestinal microbiota and related metabolic factors, in physical symptoms from the digestive tract and in intestinal permeability. In addition, we hypothesize that there are differences in other potentially relevant background factors and therefore comparisons with full siblings will give insight into family variability.

### Aim

The aim of this observational longitudinal case-control study is to compare diet, intestinal permeability, intestinal microbiota, and related metabolic factors in children and adolescents diagnosed with mental disorders and control groups to identify potential patterns that may contribute to the symptoms.

## Methods

### Study type and design

**Fig 1:**
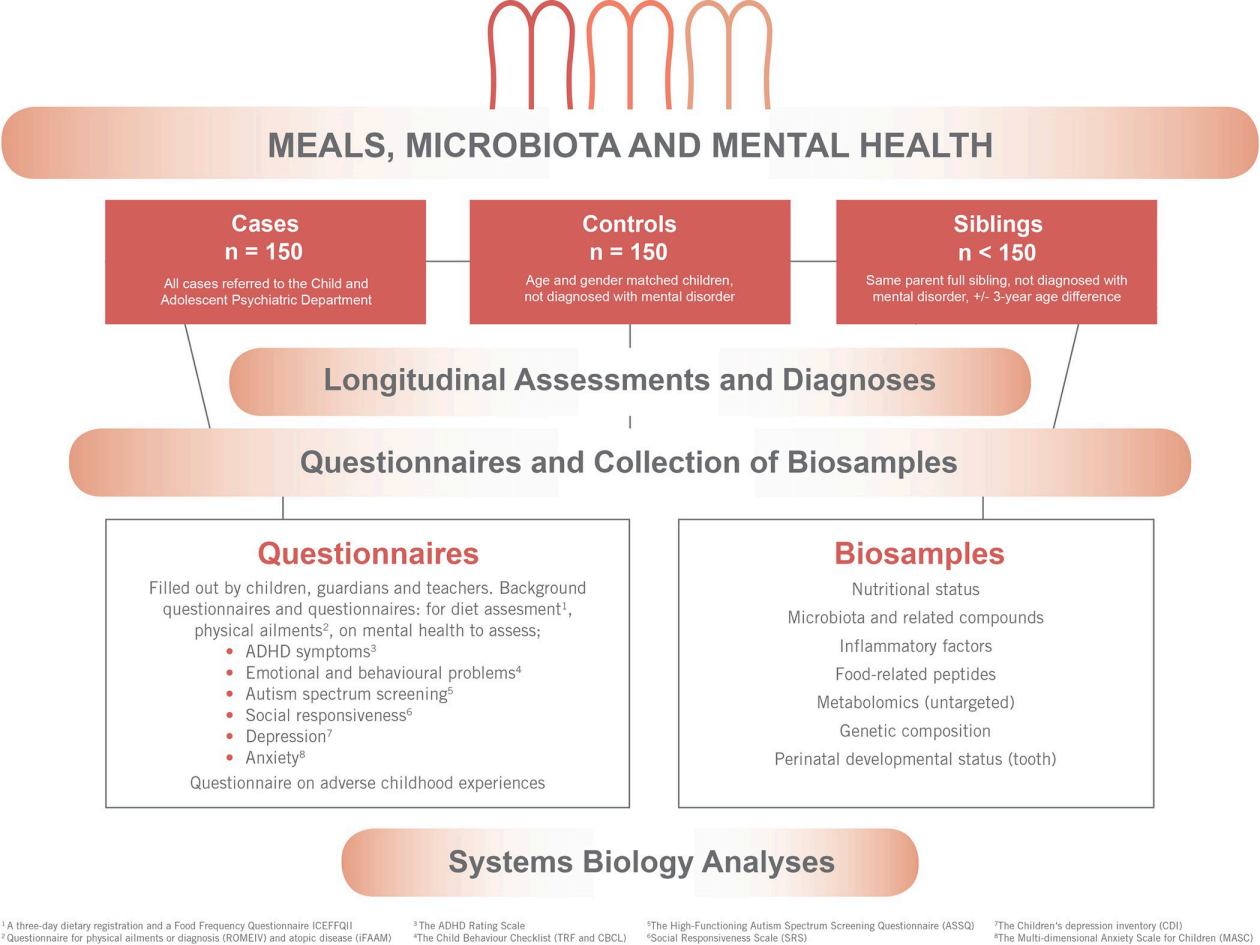
Meals, Microbiota, and Mental Health of Children and Adolescents study design.

The MMM study is a double-blinded observational longitudinal case-control study (Fig 1). Adherence to the Strengthening the Reporting of Observational Studies in Epidemiology (STROBE) guidelines was pursued throughout the study design.

#### Study population and participants, recruitment, and sample size

All children and adolescents between 5 to 15 years of age referred to the outpatient clinic of the Child and Adolescent Psychiatry Department (BUGL) over one year, will be invited to participate (*n≈*150). There are two control groups, i.e., age and sex-matched children from the same postal area (n*≈150)* and full siblings (same parent siblings, i.e., brothers and sisters) living in the same household and close in age (+/- 3 years; n<150).

#### Study group

Interested patients of the Child and Adolescent Psychiatry Department (BUGL) at The National University Hospital in Reykjavik the guardians and child will receive invitation letters with information about the MMM-study. In a follow-up phone call questions about the study can be answered and more information given. If interested to participate, an informed consent is signed, including a letter to the child’s teacher allowing the researcher to contact the teacher. For the healthy control groups (group I and II), a randomly selected child (matched by gender and age and within the same zip-code) will be contacted by the Social Sciences Research Institute (SSRI) and invited to participate similarly (control group I). Parents will be asked if a potential full sibling (+/-3 years) living in the same household could be included in the study (control group II) as we hypothesize that there are differences in other potentially relevant background factors comparisons with full siblings, and therefore, this will give insight into family variability. However, this is *not* a prerequisite for participation.

A kit including tools for collecting biosamples with good instructions and a booklet for the three-day dietary recording will be provided. In addition, links will be sent for demonstrating the specimen collection, however medical auxiliary will be available for assistance by request. Participants will be asked to be present for a blood test at the Landspitali-University Hospital in Reykjavik or Akureyri-Hospital, in Iceland, based on their residency. Eligibility criteria for the study and control groups are outlined in Table 1.

**Table 1:** Eligibility criteria for the MMM study.

Participant type	Inclusion criteria	Exclusion criteria
Cases	Children and adolescents 5–15 years, males, and females, referred for their first visit to the outpatient clinic at the Child and Adolescent Psychiatry Department (BUGL). Living in Reykjavik or Akureyri or surroundings.	Not speaking/understanding Icelandic language Recent use of antibiotics (within last 4 weeks)
Control group I: *Healthy controls*	Children and adolescents 5–15 years not diagnosed with mental disorders. Living in Reykjavik or Akureyri or surroundings.	Mental disorder diagnosis Recent use of antibiotics (within last 4 weeks)
Control group II: *Parental siblings (full siblings*, *i*.*e*., *brothers and sisters)*	Full siblings living in the same household close in age to the study subjects (+3y) not diagnosed with mental disorders. Living in Reykjavik or Akureyri or surroundings.	Mental disorder diagnosis Recent use of antibiotics (within last 4 weeks)

#### Sample size

Power calculations were performed for detecting mean difference (independent sample t-test) intestinal permeability using unmatched case-control with an ⍺ of 0.05 and β of 20% (i.e., power of 80%) with 1:1 ratio of cases vs. controls [123, 124]. The prevalence of IP among cases was calculated at 30%. However, assuming 10% prevalence in controls, the minimum number of children in each arm of the study is 62. However, as our case group covers a relatively broad spectrum of mental disorders (often overlapping individually) that will be examined in smaller case groups, the aim is set at a minimum of 100 participants in each arm (70% participation rate). The current study is an observational and non-intervention study, and therefore it is not possible to calculate power for all the factors that will be compared between the cases and controls. Corrections will be made for multiple comparisons.

### Data collection

The project has been registered by the Icelandic National Bioethics Committee (VSN19-225) in Iceland and clinicaltrials.gov (NCT04330703). The MMM-study adheres to the “Strengthening the Reporting of Observational Studies in Epidemiology” (STROBE) guidelines. All participants, i.e., cases and controls, will complete a three-day food diary, validated questionnaire on frequency of food and supplement intake according to the ICEFFQII, which is designed for the Icelandic food environment [125, 126] as well as a validated questionnaire on physical ailments or diagnoses, based on a version of the ROME IV [127–129]. Questionnaires on atopic diseases, according to the iFAAM [130–132] and medication and other essential background factors, will be completed. The mental health questionnaires used in the study have all been validated internationally as well as in Iceland. These are teacher and parent reports and self-reports for children >10 years and are listed with other questionnaires in Table 2.

**Table 2:** List of patient-answered validated mental health questionnaires and other validated and non-validated questionnaires.

	The ADHD Rating Scales [133–135]
Validated teacher and parent-reports	The Child Behavior Checklist (TRF and CBCL) [136–139]
	The High-Functioning Autism Spectrum Screening Questionnaire (ASSQ) [140]
	The Social Responsiveness Scale (SRS) [141–144]
	The Children’s Depression Inventory (CDI) [145–149]
Validated self-reports for children >10y	The multidimensional Anxiety Scale for Children (MASC) [76, 150–155]
Other validated questionnaires	Food Frequency Questionnaires (ICFFQII) [125]
	ROME IV (shorter version) [127–129]
	Questionnaires on atopic diseases iFAAM [130–132]
Other non-validated questionnaires	Questionnaires on medication and other essential background factors

Furthermore, if allowed by the guardians, information on prescription medication dispensed for the child from birth to the current date will be retrieved from the Icelandic Medicine Registry (IMR). During the recruitment period, the diagnosis of all patients coming to BUGL will be recorded to detect potential selection biases in data collection. If the recruitment is delayed, the advisory board will meet to discuss possible actions. Quality checks will be performed throughout the period, as well as simple descriptive statistical analysis. Longitudinal data collection will take place three years after the first data collection. The participants will be contacted again to collect data from both cases and controls.

#### Data management

Questionnaires are completed electronically at home using an email link (in accordance with Icelandic data privacy protection for collection and storage), with each participant having codes used for unlocking the questionnaires. These have been divided into separate sessions to minimize the burden on the case participants. The user questionnaires are available in the SmartTrial platform https://www.smart-trial.com/ (research compliant and ethics approved) and are stored at a central database collection center. This procedure has been thoroughly quality-checked by study staff and accepted for privacy and ethics approval by the ethics committee and will secure proper storage of data. Study staff will review the completed surveys within one week of receipt and highlight any missing information. Removing of data will be considered when lacking key data on diet and microbiota or over 50% of data missing. Any issues will be discussed with the principal investigator, the SmartTrial advisory team, and/or the study team. The researchers’ staff will contact the participants no more than three times either via phone, email, or in person at a regular study visit (maximum three times) to remind them to complete questionnaires and acquire answers to missing items.

### Sample collection

All biosamples, except blood, will be collected at home by the participants, with help from their guardians when needed. A biosample kit will be delivered to the participants and collected by research staff to make sure the samples will be in good condition and usable. Participants are asked to collect the biosamples preferably towards the end of the 3-day food registration within the first month into the study and store the biosamples in a specific container in a cool dry place (4–8°C) according to the manufacture’s guidelines. Commercial kits from DNA Genotek´s microbiome stool collection kits are used to collect and optimally stabilize stool samples. Fecal and saliva samples will be collected for microbiome 16s rRNA amplicon sequencing. A buccal swab will be collected for whole-genome genotyping of human DNA. Urine samples will be collected for targeted analysis of food-related peptides and untargeted metabolomics profiling. Blood will be collected either at Landspitali, University Hospital in Reykjavik, or Akureyri Hospital in Akureyri, depending on residence, for analyzing serum zonulin, inflammatory biomarkers, e.g., CRP, interleukins, and nutritional status, i.e., hemoglobin, ferritin, folic acid, Vitamin B_12_, 25-hydroxy-vitamin-D, and fatty acids. The biosamples are preferable to be collected towards the end of the 3-day food registration according to standard procedure as mentioned above. The kit is then collected from both cases and controls by the researchers along with the food diary in Reykjavik, Akureyri, and near surroundings. Participants will be able to deliver the samples to a provided location if they prefer. For other locations in Iceland, the samples will be sent out, including guidelines on how to resend them to the researchers after their collection. The urine samples will be aliquoted into smaller tubes for storing at a laboratory unit at the University Hospital or Akureyri Hospital in a -80°C freezer along with the other biological samples.

### Omics data and analyses

#### Microbiome data processing and analyses

PCR of 16S rRNA gene amplicons will be sequenced by an Illumina Miseq high-throughput platform and pair-end modality using standard protocols. Selected samples will be subjected to shotgun metagenomic library construction using the TruSeq DNA PCR-Free Library Preparation Kit by Illumina. The resulting 16S rRNA gene amplicon sequences will be analysed using the QIIME 2 microbiome bioinformatics platform [156]

For oral and intestinal microbiome data, we will perform principal coordinates analysis to investigate the compositional differences between samples. The cases and control groups will be compared concerning microbial diversity (alpha- and beta-diversity) and taxonomic abundances. Here methods developed for microbiome data to account for sparsity and under-sampling of the microbial community will be applied, such as implemented in the R package (phyloseq) [157, 158]. Correlations between microbial species will be mapped using sparCC [159], which is designed for compositional data such as microbial relative abundances and compared between case and control groups to reveal potential differences in the microbiome community structure. Shotgun metagenomic reads will be quality filtered to remove low-quality regions and screened for human reads. The taxonomic annotation will be performed using QIIME 2 [160]. Reads will be mapped against the human gut microbial integrated gene catalogue (IGC) [161]

#### Urine metabolomics processing and analyses

A morning urine sample is collected at home in a tube with peptide inhibitors. The urine is divided into two samples, one for targeted analysis of food-related peptides on an HPLC reverse phase (C-18) chromatography that gives the total peptide amount related to creatinine. The other urine sample will be used for untargeted metabolomics profiling (LC-ESI-qTOF-MS). All raw data will be extracted and processed using Agilent MassHunter Qualitative Analysis B.07.00 software. A list of peak areas, retention time and mass to charge (m/z) will be obtained and metabolites will be identified by comparing the data to selected databases. Multivariate statistical analysis will be performed using MetaboAnalyst 3.0 [162].

#### Serum measurements analyses

Concentrations of serum zonulin will be determined by ELISA assays according to the manufacturers’ protocols from Immundiagnostik AG (Bensheim, Germany) but performed at the Mucosal Immunology and Biology Research Center at the Massachusetts General Hospital in Boston. Inflammatory biomarkers will be determined by multiplex immunoassays based on the mesoscale discovery platform (e.g., CRP, interleukins) and nutritional status (hemoglobin, ferritin, folic acid, 25-hydroxy-vitamin-D, ⍵-3 fatty acids) by kits in the Landspitali laboratory in Reykjavik.

#### Nutritional analyses

For the nutritional data, the FFQs will be analyzed through PCA identifying dietary patterns in the groups. At the same time, the three-day registration will be reviewed by a dietitian and used to calculate a more exact intake of food items, calculated forward into nutrients (energy, macronutrients (protein, fat, carbohydrates/fibers)) and micronutrients (vitamins, minerals, and other bioactive compounds). Data with mean energy intake below the Basal Metabolic Rate (BER) of the child will be excluded. Furthermore, the dietary data, as well as information on the nutritional status, inflammatory factors, and omics data, will be used to study associations with oral and fecal microbiota. The same analysis will also be made in the more significant subgroups, for example, children with ADHD or ASD.

#### Genetic data and analyses

Whole genome genotyping will be performed on human DNA isolated from buccal swab samples. Genotype data on study participants will be used to investigate how host gene anchors might predict or interact with particular microbiota. Here the focus will be on genetic variants previously associated with microbiota composition or psychiatric disorders.

#### Integrative analysis of Meals, Microbiota, and Mental Health data

For each type of omics- and biomarker data (nutrients, microbiota, metabolomics, food-related peptides, inflammatory factors), appropriate preprocessing, and quality control will be coordinated and performed by the researchers. All data will be appropriately normalized and transformed, checked for potential batch effects and outliers removed. The effects of potential confounders such as age, sex, body mass index, ROME IV (for intestinal microbiota), medication, or diet will be investigated. For each datatype cases will be compared to the two control groups, but also associations between the different variables will be looked for separately, as well as in relation to sub-groups, using multivariate models including adjustments for confounder variables. Using analysis of covariance (ANCOVA) the influence of potential confounders such as age, sex, body mass index, ROME IV (for intestinal microbiota), medication and diet will be investigated. Benjamin-Hochberg false discovery rate will be applied, correcting for multiple hypothesis testing.

Integrating different types of high-dimensional omics data is a challenging task, and multiple approaches can be undertaken. A computational framework for such analysis [163] has been suggested based on dimensionality reduction of omics data using co-abundance clustering, followed by cross-omics correlation analysis focused on features that differ between cases and controls. In addition, other types of omics integration approaches will be applied, as recently reviewed [164, 165], such as robust sparse canonical correlation, co-inertia, or Procrustes analysis, to identify shared patterns across omics and dietary data in the context of mental health.

## Discussion

Investigating the interaction between diet, intestinal microbiota, IP, and their associations with mental health and comparison to healthy controls is an original and novel study. The Child and Adolescent Psychiatry Department (BUGL) at Landspitali, the National University Hospital of Iceland is the *only* psychiatric department for children and adolescents. This is the first population-based study enrolling all children with the most severe mental disorders assessed both categorically and dimensionally for one year and matched controls. This approach will provide valuable information on the relationship between these three domains offering crosstalk between different disciplines, to identify novel associations and potential mechanisms involved in neurodevelopmental disorders. There is a call for more population-based multidimensional transdisciplinary studies designed for improving mental health and our study fits well with priorities recently put forward in the Roadmap for Mental Health Research in Europe [166]. Furthermore, the World Health Organization (WHO) has recently called for a stronger focus on adolescent’s health, with depression as the number one cause of illness and disability in this age group [167]. Along with increasing the amount and precision of clinical information collected, the longitudinal approach of the MMM study will improve both reliability and validity of psychiatric diagnoses in line with LEAD (Longitudinal, Expert, All Data) procedure [168]. Furthermore, collection of multi-omics data and clinical correlates otherwise missing in this unique study population is of importance. Repeating data collection in the same groups in three years, when majority of children will be in their adolescence, gives a valuable opportunity to observe changes especially as studies on mental health are lacking in this age group [169, 170]. More multidimensional transdisciplinary studies including longitudinal observational data have been called for as a basis for lifestyle treatment options for improving mental health and wellness [169, 170]. Importantly, the longitudinal design also allows for determining if the baseline dietary, microbiome, and metabolome patterns are predictive of future outcomes, which may aid the definition of biomarker-based diagnostic tools and therapeutic interventions. The novelty of this longitudinal observational case-control study in children and adolescents resides in the new way of approaching mental disorders holistically. Using longitudinal experts diagnoses with simultaneous use of categorical and dimensional assessments using recognized and validated rating scales, identifying patterns between the leading players of meals, intestinal microbiota, IP, and mental health has never been done before. The strengths and limitations of this study are outlined in Table 3.

**Table 3:** Strengths and limitations of the MMM study.

⇒ This is the first observational study evaluating diet, microbiota, intestinal permeability, and mental health in children and adolescents in Iceland. ⇒ Recognition of the relationship between the gut-brain axis and the neuro-immune system provides a novel approach for better understanding and managing mental disorders. ⇒ More multidimensional transdisciplinary studies including longitudinal observational data have been called for as a basis for lifestyle treatment options for improving mental health and wellness. ⇒ The study will use two control groups, i.e., full siblings living in the same household close in age and age and sex-matched children from the same postal area, which is another strength of the study. ⇒ Limitations are mainly the willingness to participate as full participation, although priceless for science, is demanding. ⇒ This study will highlight associations but not causation. ⇒ Multiple variables being assessed which makes it more challenging to get at mechanisms. ⇒ Mental health molecular changes may occur years before symptoms making it difficult to determine linear relationship. ⇒ The longitudinal follow-up will help but as diet and microbiome sampling will be done 3 years out it will be even more difficult to fully state if any of the variables had an influence on mental health.

The project is a cooperation between the Unit for Nutrition Research, Faculty of Food Science and Nutrition, University of Iceland and BUGL, as well as the Department of Infectious Diseases, both at Landspítali University Hospital, but includes also national and international partners. Domestic co-operation between institutions is vital in this type of inter-disciplinary research, and all participating researchers are well-established experts in their fields. Consequently, these researchers possess complementary expertise necessary to complete the research project and answer the scientific questions put forward.

The data will give multiple possibilities for further scientific writings, as well as longitudinal data after three years. Information on publications from the study will be published on the MMM web page www.mmmrannsoknin.hi.is and on social networking sites for scientists and researchers, i.e., Akademia.edu (USA) and ResearchGate.net (Europe) and social media such as Twitter and LinkedIn. This extensive dataset will be able to answer many scientific questions and will be open to other researchers after the study period.

## Conclusions

The social *impact* of the study is high. This study will enhance understanding of gut associated contributors to mental disorders among children and adolescents in Iceland. The multi-omics approach needs to be highlighted as it is a valuable contribution. The results could increase the alternatives for treatment in mental health diseases through relevant lifestyle options. New knowledge will be obtained that could be further pursued, giving rise to new studies and influencing the quality of life and future of public health, as this group of children is vast and expanding. By diminishing symptoms and social impairment among children and adolescents, the overall school and social situation may benefit. Furthermore, increased wellbeing and decreased medical costs are of value to society.
